# Approach to signal loss in intraoperative nerve monitoring in thyroid surgery questionnaire: a Turkish surgical perspective

**DOI:** 10.3389/fendo.2025.1549988

**Published:** 2025-04-25

**Authors:** Yalin Iscan, Irem Karatas, Nurcihan Aygun, Ahmet Cem Dural, Serkan Teksoz, Ozer Makay, Ali Uğur Emre, Fatih Tunca, Mehmet Uludag, Gökhan İçöz, Yasemin Giles Senyürek, Adnan Işgör, Mehmet Haciyanli

**Affiliations:** ^1^ Division of Endocrine Surgery, Department of General Surgery, Istanbul University, Istanbul Faculty of Medicine, Istanbul, Türkiye; ^2^ Department of General Surgery, Mardin Training and Research Hospital, Mardin, Türkiye; ^3^ Division of Endocrine Surgery, Department of General Surgery, University of Health Sciences, Sisli Hamidiye Etfal Training and Research Hospital, Istanbul, Türkiye; ^4^ Department of General Surgery, Faculty of Medicine, Istinye University, Istanbul, Türkiye; ^5^ Division of Endocrine Surgery, Department of General Surgery, Cerrahpasa Medical Faculty, Istanbul University-Cerrahpasa, Istanbul, Türkiye; ^6^ Department of General Surgery, Ozel Saglik Hospital, Izmir, Türkiye; ^7^ Departmant of General Surgery, Anadolu Medical Center Hospital, Istanbul, Türkiye; ^8^ Division of Endocrine Surgery, Department of General Surgery, Ege University Hospital, Izmir, Türkiye; ^9^ Department of General Surgery, Sisli Memorial Hospital, Istanbul, Türkiye; ^10^ Departmant of General Surgery, Izmir Katip Celebi University, Atatürk Training And Research Hospital, Izmir, Türkiye

**Keywords:** thyroid surgery, recurrent laryngeal nerve, intraoperative nerve monitoring (IONM), loss of signal (LOS), staged thyroidectomy, total thyroidectomy

## Abstract

**Purpose:**

This study aimed to evaluate surgeons’ use of intraoperative nerve monitoring (IONM) during thyroidectomy and their approach to loss of signal (LOS) in various clinical scenarios.

**Materials and Methods:**

A survey was conducted by the Turkish Endocrine Surgery Society on members of the Society in February 2020 and consisted of 16 questions. The practice of IONM use, rate of inclusion in informed consent texts, and attitudes of participants in case of signal loss were investigated. The study was conducted with 183 participants between February 4-12, 2020.

**Results:**

Most participants (58.2%) had more than 10 years of surgical experience and 36.6% performed more than 50 thyroidectomies annually. IONM was routinely used by 78.7% of the participants, whereas 16.4% reserved its use in difficult cases. Only 5.2% of the participants performed continuous monitoring. In case-based LOS scenarios, the majority of participants (approximately 60%) terminated the operation when the nerve was anatomically intact but LOS persisted, except in high-risk cancer cases. When the nerve anatomy was disrupted, most participants terminated the surgery, except for the high-risk cancer group. In cases of irreversible LOS with preserved nerve integrity, 58.9% of the patients preferred continuous vagus stimulation on the contralateral side, whereas 41.1% preferred intermittent nerve monitoring. Although 68.2% of the participants verbally informed the patients about the risks of LOS, only 24.4% provided this information on the consent form.

**Conclusion:**

The use of IONM in thyroid surgery is increasing in our country. However, there is still no consensus on the approach for staged thyroidectomy in cases of signal loss, and institutional and individual differences persist. Further studies are needed to determine the medical-legal implications and effects of these variations.

## Introduction

Thyroidectomy carries a notable risk of damaging the recurrent laryngeal nerve (RLN), a complication that can significantly affect the patient’s voice function and quality of life. The RLN, which dominates the vocal box, can be damaged during thyroid surgery. This injury can result in various symptoms ranging from voice hoarseness and vocal tiredness to breathing difficulties caused by airway blockage in more extreme cases (1). The frequency of RLN damage has been inconsistently reported in the medical literature, with temporary injury rates between 5% and 8%, whereas permanent damage is estimated to occur in approximately 1–3% of procedures. Several factors contribute to the risk of RLN damage during thyroid surgery. These include the complexity of the operation, the surgeon’s experience, and variations in nerve anatomy. Research indicates that more complicated procedures such as lymph node removal or revision surgery are associated with a higher likelihood of RLN injury (2). Symptoms may differ depending on whether the injury is unilateral or bilateral. When medical-legal cases filed in our country are analyzed, bilateral RLN injuries are considered malpractice when no reason is stated for the difficulty in nerve detection in imaging or pathology reports (3).

The assessment of vocal cord function begins with preoperative vocal cord examination and continues with intraoperative RLN dissection and IONM. Identification of the RLN and dissection of the nerve up to laryngeal entry reduces the rates of RLN injury and vocal cord paralysis (VCP) and is considered the gold standard method for preventing injury in thyroid surgery (4–6). In addition to RLN identification, IONM is a valuable tool in thyroid surgery for nerve localization, identification, functional assessment, and minimization of the risk of VCP (7, 8). Continuous IONM, unlike intermittent IONM, detects adverse EMG changes indicating an impending nerve injury and allows for the modification of surgical maneuvers that could potentially cause this injury (9, 10). IONM is more preferred in thyroid cancer surgery, retrosternal goiter, and Graves’ disease, where traction injuries are more common (11).

Loss of neuromonitoring signals (LOS) during surgery signifies RLN injury, which can predict the postoperative function of the vocal cords. An IONM system showing LOS indicates a nerve conduction disorder of the RLN, such as traction, electrocoagulation around the RLN (thermal injury), or pinching (directly picking up the RLN or picking up the tissue around the nerve). The most common cause of injury is direct or indirect traction caused by tissue dissection.

During surgery, loss of the neuromonitoring signal (LOS) indicates damage to the RLN, which may predict postoperative vocal cord function. An IONM system detecting LOS suggests an RLN conduction disorder, such as traction, thermal injury from nearby electrocoagulation, or compression from direct manipulation of the RLN or surrounding tissue. The most frequent reason for this is direct or indirect traction injury resulting from tissue dissection (12).

The International Nerve Monitoring Group has provided an algorithm for managing LOS during thyroidectomy (13). However, there is an ongoing debate among endocrine surgeons and in the literature regarding the appropriateness of staged thyroidectomy after LOS on the first side for total thyroidectomy patients.

This survey aimed to evaluate surgeons’ use of IONM during thyroidectomy and their LOS approaches in various clinical scenarios.

## Materials and methods

The Turkish Endocrine Surgery Association formulated an online questionnaire comprising 16 questions using Google Sheets (Google Inc., California, USA) through an extensive review of the current literature on IONM and signal loss. The questions were structured as closed-ended, multiple-choice queries. Ethical approval was not required from the committee.

The Turkish Endocrine Surgery Association disseminated information regarding the survey through its official website and distributed it electronically to endocrine surgeons. The survey was accessible online from February 4 to 12, 2020, and received responses from 183 participants. The collected data were analyzed using Microsoft Excel, though the specific software version was not specified. The threshold for loss of signal (LOS) was set at <100 µV of amplitude on intraoperative nerve monitoring (IONM). Survey participants were endocrine surgeons managing patients with hyperthyroidism, multinodular goiter, and low- or high-risk thyroid cancer. Low-risk patients were defined as those with a tumor diameter <4 cm, unifocal disease, no extrathyroidal extension, and no lymph node or distant metastasis. In contrast, high-risk patients had a tumor diameter >4 cm, multifocal disease, extrathyroidal extension, and the presence of lymph node or distant metastases.

Participants were categorized based on their surgical experience (<5 years, 5–10 years, and >10 years), annual thyroidectomy volume (<20, 20–50, and >50 cases per year), and institutional affiliation. The survey collected data on preferred surgical approaches in thyroid cancer cases, the use of intraoperative neuromonitoring (IONM) (routine, selective, or non-use), preferred IONM modality (intermittent vs. continuous), as well as preoperative patient counseling and informed consent practices regarding LOS and nerve injury. Additionally, it assessed decision-making processes in cases of LOS, considering both the anatomical integrity of the RLN and the underlying thyroid pathology. To evaluate the impact of LOS on surgical decision-making, participants were presented with a clinical scenario involving a planned total thyroidectomy in which LOS occurred at the beginning of the procedure and did not recover. Responses were analyzed based on the underlying diagnosis (hyperthyroidism, multinodular goiter, low-risk thyroid cancer, and high-risk thyroid cancer) and whether the anatomical integrity of the nerve was preserved or disrupted. Furthermore, the survey explored participants’ monitoring preferences for the contralateral side in cases where irreversible LOS occurred on the first side despite preserved anatomical integrity.

## Results

The study participants were predominantly experienced surgeons, with 106 (58%) having more than 10 years of experience, 31 (17%) having 5-10 years of experience, and 46 (25%) having less than 5 years of experience. Regarding annual thyroidectomy volume, 67 (37%) performed > 50 cases, 55 (30%) performed between 20-50 cases, and 61 (33%) performed < 20 cases. The majority of the participants worked in academic institutions, with 116 (67.7%) in training and research/university hospitals, 25 (13.7%) in public hospitals, and 42 (23%) in private hospitals (**Table 1**).

**Table 1:** Demographic and professional characteristics of participating surgeons.

Category	Subcategory	Number of Participants	Percentage (%)
Experience Level	>10 years	106	58
	5-10 years	31	17
	<5 years	46	25
Annual Thyroidectomy Volume	>50 cases	67	37
	20-50 cases	55	30
	<20 cases	61	33
Work Setting	Training & Research/University	116	63.3%
	Public Hospitals	25	13.7
	Private Hospitals	42	23

When asked which thyroid side they started surgical procedures on in patients with thyroid cancer, the majority (91.8%) expressed a preference for the tumor-bearing side. Among the 144 participants, 78.6% utilized IONM in all cases, 30 (16.4%) reserved its use for difficult cases, and only nine (5%) did not use IONM during thyroid surgery.

After answering this question, the survey continued with 174 (95%) surgeons who used IONM. When asked which nerve monitoring technique they preferred, 133 (76.4%) participants indicated that they used intermittent IONM, 32 (18.4%) chose either continuous or intermittent IONM depending on patient characteristics, and 9 (5.2%) preferred continuous IONM in all cases.

Participants were asked to what extent they informed patients and their relatives about signal loss and nerve damage in the preoperative period and whether they included this information in the consent forms.

Forty-three (23.4%) participants reported that they provided verbal information to patients and their relatives preoperatively and obtained written consent for the use of IONM and the risk of signal loss. Additionally, 74 (40.4%) participants stated that they did not provide verbal information but obtained written consent.

While examining the approach to signal loss, participants were presented with a scenario in which they were asked what they would do if signal loss occurred at the beginning of surgery in a patient scheduled for total thyroidectomy during the preoperative evaluation and the signal loss did not recover. The questions related to this scenario were asked in two separate parts for each diagnostic group (hyperthyroidism, multinodular goiter, and low-risk and high-risk thyroid cancers), depending on whether the nerve had anatomical integrity despite LOS. In cases where the nerve was anatomically intact but the signal loss persisted, approximately 60% of the operations were terminated, except for high-risk thyroid cancer. In cases where nerve anatomy was disrupted and signal loss was observed, the majority of operations were terminated, except in the high-risk thyroid cancer group (**Table 2A**).

**Table 2A:** Rates of surgery termination in case-based loss of signal scenarios.

	RLN	
LOS (+)	Anatomical Integrity Present	Anatomical Integrity Absent
Hyperthyroidism	102 (%55.8)	149 (%81.5)
Multinodular goiter	110 (%60.1)	164 (%89.6)
Low-risk thyroid cancer	107 (%58.5)	76 (%41.5)
High risk thyroid cancer	153 (%83.7)	55 (%30.0)

The rates at which surgeons terminated surgery in the presence of LOS under different clinical scenarios were compared based on annual case volume and surgical experience. When analyzed according to surgical experience, no significant difference was observed in the rates of surgery termination across all scenarios (p > 0.05) (**Table 2B**). However, when evaluated based on annual case volume, surgeons performing > 50 cases per year were found to have a significantly higher tendency to continue surgery in high-risk cancer cases when LOS occurred and the integrity of the RLN was compromised (p < 0.005). No significant differences were detected in surgical strategies among surgeons in all other scenarios (p > 0.005) (**Table 2C**).

**Table 2b:** Comparison of surgical termination based on surgical experience in different clinical scenarios.

	Experience Level (< 5 years) (n= 46)	Experience Level (5-10 years) (n=31)	Experience Level (>10 years) (n=106)	P value
**Hyperthyroidism - RLN intact**	31 (67.4%)	17 (54.8%)	54 (%50.9)	0.61
**Hyperthyroidism - RLN not intact**	41 (89.1%)	23 (74.2%)	85 (%80.2)	0.85
**MNG - RLN intact**	35 (76.1%)	17 (54.8%)	58 (%54.7)	0.46
**MNG - RLN not intact**	40 (87.0%)	27 (87.1%)	97 (%91.5)	0.97
**Low risk cancer - RLN intact**	34 (73.9%)	18 (58.1%)	55 (%51.9)	0.45
**Low risk cancer - RLN not intact**	18 (39.1%)	12 (38.7%)	46 (%43.4)	0.92
**High risk cancer - RLN intact**	20 (43.5%)	20 (64.5%)	93 (%87.7)	0.06
**High risk cancer - RLN not intact**	9 (19.6%)	9 (29.0%)	37 (%34.9)	0.36

The bold values indicate statistical significance at p < 0.05. Chi-square test was used for analysis.

**Table 2c:** Comparison of surgical termination based on annual case volume in different clinical scenarios.

	Thyroidectomy Volume (<20 cases) (n= 61)	Thyroidectomy Volume (20-50 cases) (n=55)	Thyroidectomy Volume (>50 cases) (n=67)	P value
**Hyperthyroidism - RLN intact**	38 (62.3%)	23 (41.8%)	41 (61.2%)	0.39
**Hyperthyroidism - RLN not intact**	51 (83.6%)	43 (78.2%)	55 (82.1%)	0.96
**MNG - RLN intact**	39 (63.9%)	28 (50.9%)	43 (64.2%)	0.69
**MNG - RLN not intact**	55 (90.2%)	47 (85.5%)	62 (92.5%)	0.95
**Low risk cancer - RLN intact**	37 (60.7%)	30 (54.5%)	40 (59.7%)	0.93
**Low risk cancer - RLN not intact**	30 (49.2%)	20 (36.4%)	26 (38.8%)	0.63
**High risk cancer - RLN intact**	53 (86.9%)	50 (90.9%)	48 (71.6%)	0.64
**High risk cancer - RLN not intact**	35 (57.4%)	10 (18.2%)	10 (14.9%)	**0.0002**

The bold values indicate statistical significance at p < 0.05. Chi-square test was used for analysis.

When the participants were asked which monitoring method they preferred on the opposite side when irreversible LOS occurred at the beginning of the surgery for planned total thyroidectomy and the anatomical integrity of the nerve was preserved, 58.9% indicated that they preferred continuous vagus stimulation, whereas 41.1% preferred intermittent nerve monitoring.

## Discussion

With advances in neurophysiological monitoring techniques and increased emphasis on the protection of the RLN during surgery, IONM has become increasingly important in thyroid surgery over the last few decades. In the early 20th century, surgeons first emphasized anatomical visualization of the nerve to prevent RLN injuries based on previous knowledge. However, although injuries decreased, RLN injuries remained an important complication of thyroid surgery despite careful dissection in patients with anatomically preserved nerve integrity (14, 15). In the late 20th century, the development of electrophysiologic monitoring techniques allowed functional monitoring of the RLN during surgery (16). At the same time, the demonstration that the use of IONM in complex cases (anatomical variations, reoperations) reduced RLN injury rates contributed to the widespread use of IONM (8). In the last decade, standards for continuous and intermittent IONM techniques have been set by international guidelines advocating the need for standardized protocols to improve surgical outcomes (17).

Bilateral RLN injury is a rare but frightening complication for surgeons in patients undergoing total thyroidectomy. When this occurs, an emergency tracheostomy and subsequent reconstructive surgery may be required. Previous studies have estimated the risk of bilateral RLN injury to be approximately 0.6% (18). The widespread use of IONM facilitates the staged surgical decision-making process in patients planned for total thyroidectomy when signal loss occurs on the first side, helping prevent bilateral RLN injury (19). This complication can result from various mechanisms, such as thermal trauma, transection, ligation, clamping, traction, and compression. Traction is the most common cause of RLN injury, as identified using IONM. It has been recognized that visual assessment of the nerve’s integrity does not always accurately reflect its function, which has led to the increasing adoption of IONM in routine surgical practice. A meta-analysis comparing visualization alone with the use of IONM demonstrated that the use of IONM reduces the risk of RLN injury (20). However, there are also studies indicating that IONM does not offer superiority over nerve visualization in preventing RLN injury (21). Although it is uncertain whether IONM can decrease the incidence of postoperative RLN palsy, substantial evidence suggests that the electromyographic (EMG) response signal is a reliable predictor of postoperative vocal cord function. A normal signal indicates proper vocal cord function in 98-99% of cases (22).

The main clinical significance of intraoperative LOS detection is that it influences operative strategies such as preference for staged operative procedures to prevent bilateral RLN injuries, early prediction of VCP in the postoperative period, allowing early intervention and reducing the risk of bilateral VCP. Clinical outcomes of LOS vary depending on whether the nerve is physically preserved. Schneider et al. reported in a study that 81.7% of patients who developed LOS had vocal cord paralysis detected in early laryngoscopic examination. Permanent VCP occurred in 10.7% of patients with Type 1 LOS and 6.8% of patients with Type 2 LOS. If LOS is due to traction or neurapraxia, the likelihood of recovery is high, whereas in cases where the RLN is anatomically damaged, the probability of recovery is lower (23). Wu et al. reported that continuous IONM enhances intraoperative awareness and suggests conservative approaches if LOS is identified, which is directly linked to reduced rates of postoperative bilateral VCP (24). According to the guidelines published by the International Neural Monitoring Study Group (INMSG) in 2018, a staged surgical approach should be considered when LOS occurs on the first side, and this approach can reduce the rates of tracheostomy and bilateral VCP (17). In a survey study published by Dralle et al. in 2012, 93.5% preferred to terminate the operation after LOS was experienced at the baseline (25). In our survey, which included participants primarily composed of experienced surgeons working in academic institutions, approximately 40% opted to proceed with surgery in cases of LOS, in which the anatomical integrity of the nerve was intact, excluding patients with high-risk cancer.

In the 2020 survey conducted by the INMSG involving 950 thyroid surgeons, participants were asked how they would manage various LOS scenarios in patients planned for bilateral thyroid surgery. The majority of participants (55%-81%) preferred staged contralateral surgery when LOS occurred on the first side in cases of benign thyroid disease, although not all surgeons adopted this strategy, even for benign disease. This study highlighted the need for education on LOS management standards and guidelines during IONM decision-making processes. In the present study, when LOS occurred in malignant cases, 50.3%-72% of surgeons preferred staged thyroidectomy for low-risk papillary thyroid cancer without lymph node metastasis. However, 49.1% of surgeons indicated that they would carefully proceed with total thyroidectomy on the contralateral side in low-risk cases with lymph node metastasis, where LOS was attributed to traction injury (26). Although staged thyroidectomy is recommended in patients with LOS, some studies suggest that total thyroidectomy can be performed in these patients because LOS often improves. There is no consensus regarding this issue.

Sitges-Serra et al. showed that in patients undergoing total thyroidectomy using IONM for malignancy and multinodular goiter, in patients with LOS observed on the first side, there was 90% intraoperative LOS recovery and that bilateral thyroidectomy without bilateral nerve paralysis can be performed with caution in these cases (27).

In the study by Wu et al., which included 803 consecutive patients scheduled for preoperative bilateral thyroidectomy, staged thyroidectomy was performed in 20 of 23 patients who developed LOS on the first side, and 85% of these patients underwent completion thyroidectomy within the first six months. Total thyroidectomy was performed in three patients because of thyroid malignancy and patient comorbidities. In the postoperative period, permanent VCP was observed in two of the 23 patients, one who underwent staged thyroidectomy and one who underwent total thyroidectomy. This study highlights three options for LOS: staged thyroidectomy, safely performing subtotal thyroidectomy on the contralateral side, or proceeding with total thyroidectomy. The surgeon’s experience and underlying thyroid pathology should be considered when making these decisions (28).

Gur et al. emphasized the importance of categorizing patients based on the cause of LOS and tailoring surgical strategies accordingly. According to their study, in cases of anatomical injury (e.g., transection) where LOS is detected during dissection of the first side, the surgical indication should be reassessed. If the indications for total thyroidectomy remain valid, surgery should be performed. In such cases, intraoperative consultation with a more experienced surgeon from the same institution is recommended and contralateral surgery should be completed using continuous IONM. If this option is unavailable, the procedure should be halted and the patient should be referred to a specialized center. In scenarios where the RLN is anatomically intact, staged thyroidectomy should be preferred regardless of the initial indication (29).

Ramesh et al. stated that total thyroidectomy should be considered in the same session in patients who are planned for total thyroidectomy and develop a first-sided LOS in patients with urgent reasons and in patients who may expect surgical difficulties in secondary surgery (30).

In our survey, approximately 60% of the respondents preferred staged thyroidectomy for benign thyroid disease and low-risk thyroidectomies when the nerve was intact, while 40% reported that they would continue with the surgical procedure. The preference for staged thyroidectomy has increased to 80% of high-risk thyroidectomies. When the nerve was not anatomically intact, the preference for staged thyroidectomy exceeded 80% in benign cases, 40% in low-risk thyroidectomies, and 30% in high-risk thyroidectomies.

In our study, there were no data on how many of these cases involved bilateral LOS or whether the loss resulted in bilateral VCP. Therefore, the medicolegal implications of this approach remain controversial. Nonetheless, this finding indicates that visual identification of the RLN and the perception that its anatomical integrity ensures functional preservation remain prevalent.

In a survey conducted in Turkey between December 2016 and January 2017, the aim was to assess the attitudes of thyroid surgeons toward approaches used to avoid or manage voice and airway complications during surgery, and it was found that the rate of IONM use was 36% (31). Similarly, in our study, it was observed that awareness of IONM usage among endocrine surgeons in our country has increased, with the rate being nearly three times higher than that four years ago. Notably, 95% of the participants preferred IONM in all cases. The high rate of IONM use in our survey is similar to the results of an international survey (32). However, in contrast to the results of this study, the preference for continuous IONM was lower (5.2% vs. 21.1%). A meta-analysis summarizing ten reviews indicated that the use of IONM in challenging cases, such as advanced cancer surgery, recurrent patients, and plunging goiter, resulted in a decrease in RLN paralysis rates. In our study, 16.4% of the participants preferred to use IONM only in challenging cases rather than routinely.

Our study had certain limitations. First, the survey participants were selected from members of the Turkish Endocrine Surgery Association, with the majority comprising surgeons who had been working in experienced centers specializing in endocrine surgery for many years. This may not fully reflect the national perspective and practices regarding IONM use and surgical strategies in the presence of LOS. There are differences in experience and current approaches, even among surgeons working in high-volume institutions in Turkey. Moreover, individual experience, apart from institutional practices and surgical volume, may influence surgical approaches and intraoperative decision-making by surgeons.

In conclusion, the use of IONM in thyroid surgery is increasing in our country. However, the inclusion rate for preoperative informed consent was low. There is still no consensus on the approach for staged thyroidectomy in cases of signal loss and institutional and individual variations exist. Further studies are needed to determine the medical-legal implications and effects of these differences.

## Data Availability

The original contributions presented in the study are included in the article/supplementary material. Further inquiries can be directed to the corresponding author.
